# Clinical Relevance of HLA Antibody Monitoring after Kidney Transplantation

**DOI:** 10.1155/2014/845040

**Published:** 2014-10-13

**Authors:** Christian Morath, Gerhard Opelz, Martin Zeier, Caner Süsal

**Affiliations:** ^1^Department of Nephrology, University of Heidelberg, Im Neuenheimer Feld 162, 69120 Heidelberg, Germany; ^2^Department of Transplantation Immunology, Institute of Immunology, University of Heidelberg, Im Neuenheimer Feld 305, 69120 Heidelberg, Germany

## Abstract

In kidney transplantation, antibody-mediated allograft injury caused by donor HLA-specific antibodies (DSA) has recently been identified as one of the major causes of late graft loss. This paper gives a brief overview on the impact of DSA development on graft outcome in organ transplantation with a focus on risk factors for *de novo* alloantibody induction and recently published guidelines for monitoring of DSA during the posttransplant phase.

## 1. Introduction

The employment of new immunosuppressive strategies in the late 1980s and 1990s and their efficacy to control T-cell alloimmunity led to a striking decrease in the occurrence of T-cell-mediated acute rejection episodes. Simultaneously, our shortcomings in controlling antibody-mediated rejection (AMR) were revealed and the importance of chronic AMR has become apparent. Mainly two developments contributed to a deeper understanding of antibody-mediated allograft injury: (1) the recognition that deposition of the complement split product C4d (especially in peritubular capillaries of the kidney allograft) may indicate antibody-mediated allograft injury and (2) the association of donor HLA-specific antibodies (DSA) detected by highly sensitive techniques with inferior outcome of kidney transplants [1, 2]. Recent investigations indicate that more than 60% of late kidney graft losses are due to antibody-mediated humoral tissue injury, and there has been increasing evidence that HLA antibodies are responsible for graft losses not only in kidney but also in other solid organ transplantations [3–5]. Therefore, HLA antibodies and their association with AMR have become the main focus of research in organ transplantation.

## 2. Tissue Damage Caused by Donor HLA-Specific Antibodies

Early after transplantation, acute AMR occurs in about 1 to 6% of patients; however, this frequency may increase up to 21 to 55% in patients who had detectable DSA already before transplantation and who received desensitization therapy [6–8]. Persistence or reemergence of DSA that were detectable already before transplantation is associated with poor allograft outcome [9]. Weak pretransplant DSA have been associated with rather subtle types of graft damage, often leading to delayed graft function [10]. It is well known that early damage can later on translate into chronic (antibody-mediated) changes, most likely because the structure of the endothelium is injured and new antigenic epitopes are expressed on the surface of transplanted tissue. During later phases after transplantation, insufficient immunosuppression and stimulation of the memory cell response by inflammatory events can support the development of* de novo* DSA against antigenic structures and result in failure of the transplanted organ due to antibody-mediated organ injury.

Additional antibodies that are discussed in the evolution of chronic AMR are MICA antibodies, angiotensin II type 1 receptor activating antibodies, and other antiendothelial cell antibodies [11–13]. The exact impact of these antibodies on the outcome of kidney and other organ transplants needs, however, yet to be determined.

In this overview, we focus on the impact of* de novo* HLA alloantibodies that are detected after kidney transplantation.

## 3. Donor HLA-Specific Antibodies Become the Most Important Parameter in the Diagnosis of Antibody-Mediated Kidney Allograft Rejection

Currently, features of AMR in the biopsy together with the detection of a circulating DSA are required for the histological diagnosis of antibody-mediated kidney graft rejection. In addition, evidence of antibody interaction with the vascular endothelium must be present, either by C4d positivity or microvascular inflammation (peritubular capillaries and/or glomerulitis) [14]. Of note, in the latest update of the BANFF classification (BANFF 2013), detection of C4d-positivity in peritubular capillaries is no longer considered a prerequisite for the diagnosis of AMR. Instead, moderate microvascular inflammation or even the demonstration of AMR-specific gene transcripts together with circulating DSA is accepted as diagnostic criterions for the diagnosis of AMR. In particular, in chronic AMR, C4d may often be negative (C4d-negative AMR). Before the introduction of highly sensitive antibody detection techniques, such as the Luminex single antigen bead (L-SAB) assay, there was often no DSA detectable in patients with chronic AMR due to the low levels of antibody. L-SAB now allows the detection of DSA with high sensitivity. Only recently, Wiebe et al. reported that even weakly reactive, L-SAB-detected* de novo* DSA measured at the low positivity cut-off of 300 MFI is predictive of graft survival [15]. Everly et al. confirmed this observation, with the exception that they used the higher cut-off of 1,000 MFI [16].

## 4. Risk Factors for the Development of (*De Novo*) Donor HLA-Specific Antibodies

Risk factors for the development of* de novo* DSA, AMR, and graft loss are not uniformly described. In many patients with late antibody-mediated graft loss, even when HLA class I alloantibodies are detectable, circulating HLA class II* de novo* DSA are considered to be mainly responsible for rejection. Therefore, most authors believe that specifically HLA class II mismatches (not only HLA-DR but also HLA-DQB, DQA, and DP mismatches) confer an increased risk for late graft loss [15–17]. Due to the strong linkage disequilibrium between the DR and DQB or DQA (but not DP) gene loci, two DR mismatches often indeed represent 6 mismatches that are relevant for induction of DSA. Additional risk factors for the* de novo* development of DSA and subsequent occurrence of (chronic) AMR are younger age, deceased donor kidney transplantation, presence of HLA antibodies before transplantation, nonadherence to immunosuppressive medication (see below), and insufficient immunosuppression or drug minimization [4, 16, 17].

Most importantly, early inflammatory events, such as infections, minor surgery, trauma, and particularly early (acute) T-cell-mediated rejection episodes, often precede* de novo* DSA development and AMR [18–20]. Even subclinical cellular rejection may lead to HLA antibody development with an increased risk for antibody-mediated allograft injury in subsequent years [7].

Table 1 gives an overview on the risk factors for* de novo* DSA development.

## 5. Graft Survival after Development of* De Novo* Donor HLA-Specific Antibodies

Hidalgo et al. found DSA in 37% of patients who had an indication biopsy 7 days to 31 years after transplant [23]. In particular,* de novo* DSA, which made up 60% of all DSA and were directed against HLA class II antigen mismatches of the donor, were associated with strongly impaired graft survival: within 5 years from DSA detection, 50% of the patients in the study of Hidalgo lost their grafts. Wiebe et al. found a 10-fold increase in graft loss in patients who developed* de novo* DSA, with a 40% lower graft survival rate 10 years after DSA development compared to patients without* de novo* DSA [15]. Everly et al. reported on a 24% graft loss rate 3 years after* de novo* occurrence of DSA [16]. However, in all these studies, low numbers of patients with graft loss were investigated. We compared in sera of 51 patients with graft loss that were obtained prior to graft failure and in sera of matched controls with functioning grafts the incidence of* de novo* DSA and non-DSA [24]. Patients with graft loss showed a higher incidence of both DSA and non-DSA than patients without graft loss.

## 6. C1q-Binding HLA Antibodies

A recent development is the introduction of solid-phase assays that allow the distinction of complement-binding (C1q assay) or complement-activating (C4d assay) HLA antibodies from HLA antibodies that do not bind or activate complement.

While two early pediatric studies that investigated the use of the C1q assay for the detection of* de novo* DSA after transplantation found conflicting results [25, 26], a recent landmark study by Loupy et al. demonstrated that the occurrence after transplantation of complement-binding DSA in a cohort of 1,016 patients transplanted between January 1, 2005, and January 1, 2011, was associated with adverse outcomes [21]. The 5-year graft survival rate in patients who developed complement-binding DSA (*de novo* and persistent/reemerging) was 54%, strikingly lower than the 93% rate in patients with DSA that were not complement-binding or the 94% rate in patients without any DSA. The higher graft loss rate in patients with complement-binding DSA was attributable to a higher rate of AMR, especially in the patients who developed complement-binding DSA* de novo* after kidney transplantation. Interestingly, pretransplant complement-binding DSA did not have the same predictive values since about half of the patients lost these antibodies after transplantation.

## 7. Nonadherence and Reduction of Immunosuppression as Major Contributors to Late Graft Loss

Einecke et al. reported in 2009 that antibody-mediated microcirculation injury is one of the leading causes of late graft loss, together with death with a functioning graft, recurrent renal disease, and interstitial fibrosis/tubular atrophy (of unknown origin) [3]. Chronic AMR is found more frequently in patients who are nonadherent to immunosuppressive medication or in whom immunosuppression was reduced or withdrawn for other reasons, for example, conversion to calcineurin-inhibitor-free or steroid-free immunosuppressive protocols, recurrent infection, or malignancy [4, 15, 17, 21]. Patients at high risk for nonadherence are young adults who are in the transition phase from pediatric to adult renal services. Other risk factors are previous nonadherence, psychiatric disorders, substance abuse, and insufficient socioeconomic support, but also adverse effects of immunosuppressive medication.

In a recent publication, 64% of graft losses in a selected patient cohort with indication biopsies were found attributable to (antibody-mediated) rejection [4]. Importantly, about half of the patients with rejection-associated graft loss were identified as nonadherent. In the study of Wiebe et al., who investigated the evolution of HLA antibodies after transplantation,* de novo* DSA were found to appear at a mean of 4.6 years after transplantation, and the prevalence of* de novo* DSA after 10 years was 20% in adherent as compared to a remarkable 60% in nonadherent graft recipients [15]. Of note, in this study, patients were thoroughly screened to exclude any preexisting antibodies. Patients were considered to have* de novo* DSA only when posttransplant DSA occurred after the current and all historic sera at the time of transplantation were negative at a 300 MFI cut-off and when patients showed no evidence of AMR in a 6-month protocol biopsy. With this definition, no* de novo* DSA were detectable at 6 months and only a 2% incidence of* de novo* DSA was recorded at 1 year.

Not only nonadherence of the patient to immunosuppressive medication but also reduction of immunosuppression by the physician may lead to adverse outcomes after kidney transplantation. Opelz and Döhler hinted already in 2008 on the problem of “insufficient immunosuppression” as a major cause of graft loss. In an analysis of more than 25,000 kidney transplant recipients, they showed that, in patients with good graft function at year one, discontinuation or reduction after the first posttransplant year of cyclosporine, tacrolimus, or mycophenolate mofetil below certain threshold levels was associated with significantly reduced graft survival during the subsequent years [27].

Insufficient immunosuppression may also occur during immunosuppressive minimization (tapering) or calcineurin-inhibitor-avoidance trials. In a recent study, 14 of 61 patients (23%) that were converted from cyclosporine to everolimus at 3–4.5 months after transplantation developed DSA, compared to only 7 of 65 patients (11%) who continued on cyclosporine [17]. Eight patients on everolimus, but only 2 patients on cyclosporine, developed AMR. It needs to be mentioned that in this study many patients off cyclosporine, unfortunately, were also off steroids which might have biased the results. Nevertheless, these data indicate that particularly patients with reduction or discontinuation of immunosuppressive medication should be screened rigorously for the occurrence of HLA antibodies and antibody-mediated allograft injury. In patients with DSA, minimization of immunosuppression should altogether be avoided. Nonadherence to immunosuppressive medication and insufficient immunosuppression does not only lead to the development of* de novo* DSA but has also significant impact when DSA already are present [15]. Wiebe et al. found nonadherence in 100% of patients with* de novo* DSA and acute graft dysfunction, whereas the rate was only 6% in patients with* de novo* DSA but stable graft function.

## 8. What Do the Guidelines of the Transplantation Society (TTS) Tell Us about Posttransplant Antibody Monitoring?

Several important issues are covered by the consensus guidelines that have been published in early 2013 [28].

(1) Posttransplant screening for DSA is recommended for all patient groups in the early postoperative period, however, at different time points dependent on the pretransplant risk of the patient for AMR. In “low risk” patients, who were not sensitized to HLA before transplantation and who received their first allograft, the possible presence of DSA should be examined at least once in the period from 3 to 12 months after transplantation. In “intermediate risk” patients, who were antibody-negative at the time of transplantation but had DSA in previous testing, DSA should be examined already during the first month. No further testing is recommended for both groups during the first year, unless (i) there is a change in immunosuppression, (ii) nonadherence is suspected, (iii) graft dysfunction occurs, or (iv) the patient is transferred to a remote outside center. (2) If DSA are present at any time, a biopsy should be performed, and if the biopsy result is positive, treatment of AMR is recommended. In DSA-positive “high risk” patients and in desensitized crossmatch-positive “very high risk” patients, in addition to DSA monitoring, a biopsy is recommended for all patients during the first 3 months after transplantation. Even if the biopsy result is negative in these two groups but there are rapidly increasing DSA or if the biopsy shows subclinical rejection, treatment of AMR should be initiated. In the absence of AMR, DSA should be monitored and immunosuppression maintained at higher levels. (3) Beyond year one, no routine DSA monitoring is recommended for the four risk groups, except when one of the abovementioned four conditions occurs. Of note, a minority of members within the guidelines group supported HLA antibody monitoring at least once a year in all patients to rule out antibody-mediated allograft injury at its earliest stage. (4) If DSA are detected beyond year 1, patients should be treated and monitored essentially as described above for the first year after transplantation.

It needs to be mentioned that the consensus guidelines of TTS were published before Loupy and coworkers published their seminal paper on the impact of complement-binding HLA alloantibodies on kidney graft survival.

## 9. Conclusions

In summary, despite its known technical limitations [29], the highly sensitive L-SAB assay appears to be a very useful tool for posttransplant monitoring of HLA antibodies and for surveillance of AMR. HLA antibodies that occur* de novo* after transplantation and that are complement-binding/activating denote the highest risk for AMR and graft loss. However, also the recurrence of preexisting antibodies after transplantation or the development of* de novo* non-DSA may confer an increased risk for graft loss.

## Figures and Tables

**Table 1:** Risk factors for the *de novo* development of DSA.

Risk factor	Reference
Retransplantation	[21]
HLA antibodies before transplantation	[16, 21]
Young age (18–35 years old)	[16]
Deceased donor transplantation	[16]
DR, DQ mismatch	[16, 22]
Nonadherence	[4, 15]
Insufficient immunosuppression	[17]
Inflammation (i.e., infection)	[18, 19]
(Subclinical) T-cell-mediated rejection	[7, 15]
